# Haven Ley: health and the empowerment of women

**DOI:** 10.2471/BLT.24.031224

**Published:** 2024-12-01

## Abstract

Haven Ley talks to Gary Humphreys about Pivotal’s plans to address women’s health, including sexual and reproductive rights, as part of broader efforts to empower women.

*Q: Pivotal Ventures recently announced a 1 billion United States dollars (US$) investment in advancing women’s power. US$ 250 million of that money is be allocated through a worldwide open call for innovative initiatives focused on women’s health, including sexual and reproductive health. What was the thinking behind these initiatives?*

A*: *Melinda French Gates started Pivotal Ventures 10 years ago as a complementary organization to the Bill & Melinda Gates Foundation, focused on social progress in the United States. From the beginning, we knew we would have a focus on women and gender equality, and over time we refined our vision to expand women’s power and influence in key sectors and reduce barriers to their participation in society. This was something I was able to contribute to, having had experience developing and managing a gender strategy and helping to seed the early years of the gender programme at the Bill & Melinda Gates Foundation, which continues to this day. Since then, Pivotal has funded a number of gender-focused initiatives across key focus areas, including expanding women’s political power, building a modern caregiving system and increasing women’s participation in the tech industry. Notably, Pivotal launched the Equality Can’t Wait Challenge in June 2020 aimed at funding bold ideas for advancing gender equality, offering US$ 40 million in grants to organizations with transformative plans to expand women’s power and influence in the USA by 2030.

*Q: How does Pivotal target its resources?*

A: Pivotal uses a model that leverages three kinds of capital: philanthropic, investment and political. We look at the specific role of philanthropic capital as really trying to supplement where there’s been under-investment in certain aspects of women’s health or forgotten marginalized populations. In terms of venture capital, Pivotal engages in venture funds and direct investments in projects, particularly those involving women-led funds and companies. This approach aims to prove that investing in diverse founders can yield meaningful financial returns while promoting market-based solutions to social problems. By committing early to these ventures, Pivotal helps attract additional investments that can drive growth and sustainability​. On the political front, Pivotal seeks to promote policies that support women’s empowerment by collaborating with various stakeholders to advocate for systemic changes at local, state and national level.

“Women’s control over their bodies […] is foundational.”

*Q: At what point did you expand the scope of the initiative to take in the global community?*

A: When Pivotal was created, there was tremendous demand for more resources to address gender inequalities in the United States, especially regarding women and girls of colour, so that’s where we focused our work. But a global perspective has always been baked into our thinking. Melinda has 25 years of experience as a global philanthropist, and I had previously worked in low- and middle-income countries, including working with state-funded programmes in East Africa and Mongolia. When Melinda announced her departure from the Foundation in May 2024, she asked that we deepen our work to include a focus on women’s health as core to expanding women’s power, and that we apply our impact model to the global context.

*Q: When did you decide to include considerations of women’s health as part of the empowerment challenge?*

A: Women’s health has always been a part of our considerations regarding women’s economic empowerment and leadership. However, I think it’s fair to say that a significant turning point came in 2022 when Roe v. Wade (a Supreme Court decision establishing a woman's legal right to choose to have an abortion) was overturned. At that point, Pivotal began to explore where we could expand our purview and we began investing in women’s health, as an integral part of considerations of women’s empowerment and leadership. This work has only increased. Most recently, as you noted, Melinda committed to invest US$ 1 billion in women’s power around the world, including US$ 250 million in the “Action for Women’s Health” open call, launched in October 2024. The global open call was designed to support organizations working to improve the mental and physical health of women and girls worldwide. The initiative is entirely philanthropic, and we are currently focusing on nonprofit and community-based organizations with innovative women's health interventions that require charitable capital. Despite significant funding for sexual and reproductive health, many areas remain neglected. Issues like ageing and menopause rarely receive attention or significant funding, for example.

*Q: How does access to sexual and reproductive health services tie into the broader issue of economic empowerment for women?*

A: In our view, sexual and reproductive health is foundational to women’s economic empowerment. It underpins women’s ability to work, pursue education, and make life choices that can enhance economic stability. Access to reproductive health care, including contraception, maternal health services and safe childbirth, allows women to better control family planning, reducing interruptions in education and employment. This, in turn, strengthens their economic security and participation in the workforce. Several studies have shown that access to contraception increases women’s earning potential by enabling them to complete higher education and obtain higher-paying jobs, which fosters economic independence​. Furthermore, addressing maternal health and reducing preventable health issues, such as complications from childbirth, not only supports individual well-being but also mitigates significant financial burdens for women, their families, and health-care systems. Improving reproductive health access has been linked to lower poverty rates and increased household incomes as women invest more time and resources in personal and familial growth​.

This alignment of health and economic empowerment makes reproductive health initiatives essential for advancing gender equality and economic resilience at individual and community levels. Our feeling is that by addressing these areas through our work and giving, we can significantly enhance women’s power and influence across a range of sectors. Also, speaking personally, I think that we’d be missing a trick if we didn’t think about access to care as the one thing that women and girls have – or should have – complete control over; that is to say, their bodies and their health, including their emotional and mental health. For me, women’s control over their bodies and their health is foundational to the entire empowerment agenda.

“Issues like ageing and menopause rarely receive attention.”

*Q: There are different views regarding access to sexual and reproductive health services both in the USA and around the world. To what extent does Pivotal take account of these differences as it engages with different cultures and countries?*

A: As a student of gender analysis and norms, cultural nuances are always a consideration in my work, and this also true of Pivotal. We will also be drawing on relevant research and, of course, Melinda’s 25 years of experience working on this issue. Anything that Pivotal decides to do in this space will be informed by listening to and collaborating with community leaders. Indeed, community-based support for the interventions that are being proposed will be considered a key criterion for success, as well as for the health and safety of any beneficiaries. Moreover, Pivotal won’t be advocating for interventions directly, but will support certain community-based organizations who hopefully have buy-in from the communities in which they're working to promote and support choice for young women.

*Q: What sort of projects are you hoping to see?*

A: The open call is aimed at catalyzing initiatives that increase women’s access to health, fundamentally. We have designed the competition to be as broad as possible, and want to remain really open regarding the trends and the opportunities that are out there. As a funder, I am also really interested in the gaps where there are no interventions right now and where I think that there could be with additional attention and support. We are hoping to support up to 100 initiatives. The second major objective is to shine a spotlight on investable ideas that are already out there and that are poised to scale. So, in addition to the capital that we invest, we’ll be working to make sure that other funders are seeing the kinds of projects that meet our criteria.

*Q: You referenced your use of political capital. How do you do that without stepping on the toes of elected officials?*

*A:* We do it very carefully and mindfully within the confines of the guidelines used by the US government, and we always work in careful partnership. In the United States, change happens when you have policy-makers and decision-makers advocating on the issues that people care about, whether that’s a caregiving economy that works for all, or helping more women run for and succeed in elected office. We have had a lot of success partnering with influential leaders – champions – who advocate for social progress, gender equality and other causes, and we’re exploring how to apply this model globally, recognizing that we may need to adjust our approach according to the specific contexts we operate in. In fact, as part of her US$ 1 billion commitment, Melinda announced that she would provide US$ 20 million to 12 champions who can then direct the money toward organizations of their choice, targeting areas like reproductive health, racial equity and gender equality. This model not only provides funding but also leverages each leader’s influence to raise awareness, create momentum for grassroots efforts and inspire broader systemic change in communities.

*Q: Are any of the champions targeting sexual and reproductive health?*

A: Yes, they are or, we believe, will be. For example, Allyson Felix, the most decorated female Olympian in track and field, uses her platform to advocate for black maternal health and pregnancy protections among other issues.

*Q: When will you be announcing the successful open call candidates?*

A: Registration for the open call closed in early December and we expect to announce awardees at the end of 2025. The vetting process is being handled by Lever for Change, a nonprofit affiliate of the MacArthur Foundation. They've created this incredibly robust, transparent and inclusive process, but it takes about a year to work through. We’ll go into the submission and judging phase over the next nine months. So, watch this space.

